# Associations between Frequency of Culinary Herb Use and Gut Microbiota

**DOI:** 10.3390/nu14091981

**Published:** 2022-05-09

**Authors:** Alexandra Adorno Vita, Ryan McClure, Yuliya Farris, Robert Danczak, Anders Gundersen, Heather Zwickey, Ryan Bradley

**Affiliations:** 1 Helfgott Research Institute, National University of Natural Medicine, Portland, OR 97201, USA; agundersen@nunm.edu (A.G.); hzwickey@nunm.edu (H.Z.); rbradley@nunm.edu (R.B.); 2 Biological Sciences Division, Pacific Northwest National Laboratory, Richland, WA 99352, USA; ryan.mcclure@pnnl.gov (R.M.); yuliya.farris@pnnl.gov (Y.F.); robert.danczak@pnnl.gov (R.D.)

**Keywords:** microbiota, culinary herbs, phytochemicals

## Abstract

While evidence suggests that culinary herbs have the potential to modulate gut microbiota, much of the current research investigating the interactions between diet and the human gut microbiome either largely excludes culinary herbs or does not assess use in standard culinary settings. As such, the primary objective of this study was to evaluate how the frequency of culinary herb use is related to microbiome diversity and the abundance of certain taxa, measured at the phylum level. In this secondary data analysis of the INCLD Health cohort, we examined survey responses assessing frequency of culinary herb use and microbiome analysis of collected stool samples. We did not observe any associations between frequency of culinary herb use and Shannon Index, a measure of alpha diversity. Regarding the abundance of certain taxa, the frequency of use of polyphenol-rich herbs and herbs with certain quantities of antibacterial compounds was positively associated with Firmicutes abundance, and negatively associated with Proteobacteria abundance. Additionally, the total number of herbs used with high frequency, defined as over three times per week, was also positively associated with Firmicutes abundance, independent of adjustments, and negatively associated with Proteobacteria abundance, after adjusting for dietary factors. Frequency of culinary herb use was not associated with Bacteroidota or Actinobacteria abundance.

## 1. Introduction

The gastrointestinal (GI), or gut, microbiome is highly diverse and includes microbiota from more than 1000 species of bacteria, as well as some archaea, protists, viruses, and fungi. The most predominant residents, however, are bacteria from two major phyla—Firmicutes and Bacteroidetes—which comprise over 90% of the microbiota [1,2,3]. While every individual’s microbiome is unique, certain predominant “enterotypes” have emerged, each characterized by its own distinctive compilation of microbiota and dominant genera [4,5]. The gut microbiota and their downstream products (e.g., metabolites, synthesized vitamins, amino acids, and neurotransmitters, etc.) directly affect the regulation of immune responses, coordination of neuroendocrine and cardiovascular activities, facilitation of host nutrition and metabolism, and maintenance of intestinal barrier function [3,6]. Due to these wide-ranging physiological effects, it is perhaps unsurprising that recent evidence highlights a role for the gut microbiome in the pathophysiology of numerous inflammatory, metabolic, and neurological diseases [7]. More specifically, the generation and severity of many such diseases have been linked to dysbiosis of the microbiome, which is characterized by the increased and/or decreased abundance of specific microbes and their metabolites to an extent that disrupts homeostatic conditions within the body [7].

### 1.1. Diet and the Microbiome

While there are numerous intrinsic and extrinsic variables impacting microbial composition within the gut, diet has emerged as one of the predominant influencing factors that is also modifiable, and thus has therapeutic potential. The ability of diet to shape microbial communities, both long-term and short term, is largely in response to available macro and micronutrients [8]. Macronutrients (i.e., proteins, fats, complex carbohydrates including fiber) can either selectively promote or inhibit growth, depending on the preferred and available metabolic pathways of specific bacteria, as well as microbial sensitivity to macronutrient-induced changes within the gut, such as bile acid production and shifting pH [5,8]. Additionally, micronutrients (i.e., vitamins and minerals) and other physiologically relevant plant-derived bioactive compounds (i.e., secondary metabolites, such as polyphenols, alkaloids, terpenoids, etc.) can impact microbial metabolism, enzyme function, and gene expression [9,10,11,12]. Additionally, certain macronutrients, such as fiber [8], and certain micronutrients, such as polyphenols [13], act as energy sources for bacterial growth, and are considered prebiotics.

### 1.2. Culinary Herbs and Spices

Culinary herbs and spices contain micronutrients and bioactive compounds (e.g., polyphenols, alkaloids, etc.) [11,14,15], yet have not been widely studied regarding their impact on the GI microbiome in human populations, and thus represent a confounder in research investigating diet and microbiota. There is much pre-clinical evidence regarding the impact of culinary herbs and spices, or their active constituents, on GI microbiota [12,16,17,18,19]. However, these often do not utilize the herbs and spices (or their active constituents) in doses, combinations or frequency of administration relevant to typical culinary use. In human samples, some herbal mixtures, containing culinary herbs and spices, have been used as interventions in clinical investigations, such as a single capsule of mixed curry spices [20], a single capsule of mixed culinary spices (cinnamon, oregano, ginger, black pepper, and cayenne pepper) [21], and an herbal formula containing both culinary spices and specific nutrients [22], among others. There have also been evaluations of specific herbs and/or known active constituents on the microbiome (e.g., oregano [23], capsaicin [24], curcumin [25], etc.,). Many report findings in which the abundance of beneficial bacteria increase while that of opportunistically pathogenic bacteria decrease. While such studies may help elucidate a causal relationship between the use of specific herbs and changes in microbiota, and are thus important, they are not representative of how these products are consumed during typical cooking practices.

### 1.3. Research Objective

Due to the emerging role of the microbiome in health and disease [26], along with existing evidence that culinary herbs have the potential to modulate gut microbiota, evaluating associations between cooking with herbs and the gut microbiome may be hypothesis-generating for future experimental research, and provide insight into prospective therapeutic strategies. As such, the primary objective of this study is to evaluate if the frequency of culinary herb use is associated with microbiome diversity and abundance of certain phyla.

## 2. Materials and Methods

### 2.1. Study Design

This study is a secondary analysis of data collected during the INCLD Health longitudinal cohort study, the methods of which have been previously published [27]. Briefly, data from participants was collected over the course of three time points—baseline, 6 months, and 1 year—and included various evaluations of health, wellness, lifestyle, diet, and the microbiome. In this ancillary study, only select data from the baseline visit was used. Survey data was collected and managed using REDCap [28] electronic data capture tools hosted at the National University of Natural Medicine. Nutritional data was collected with VioScreen (by VioCare, Princeton, NJ, USA), a validated food survey that was administered online, and calculated by the Nutrient Coordinating Center.

### 2.2. Participants

Participants in the original study were students enrolled in a complementary and integrative medicine education program and were previously recruited through methods outlined in the INCLD Health protocol [27]. While data was collected from 197 participants during the INCLD Health study, data from only 96 of those participants were used in the statistical analyses of this ancillary study for reasons outlined under the “*Statistical models*” sub-section. The final working sample was roughly 15% male and 85% female, with an average age of 29. About 75% of the sample was White/Caucasian, 5% Asian, 2% Black/African American, 2% Middle Eastern, 1% Native Hawaiian/Pacific Islander, 1% Native American/Alaskan, 6% Mixed, and 4% other/unknown.

### 2.3. 16S rRNA Gene Sequencing and Processing

All 16sRNA gene sequencing and processing was carried out at the Pacific Northwest National Laboratory (Richland, WA, USA). DNA was extracted from participant fecal samples using the Quick-DNA Fecal/Soil Microbe Microprep Kit (Zymo, Irvine, CA, USA). The hypervariable V4 region of the 16S rRNA gene was sequenced on an Illumina MiSeq using the 515F-806R primer set. The resulting 16S rRNA amplicon dataset was processed using QIIME2 (v2021.4) [29]. Within the QIIME2 environment, DADA2 (*q2-dada2*) [30] was used to both denoise and cluster amplicon sequence variants (ASVs), which were then taxonomically classified (*q2-feature-classifer*) using the SILVA database (v138) [31]. Processed data was then exported from QIIME2 and converted into a comma-delimited file.

### 2.4. Microbial Ecology Analyses

Ecological analyses were performed using the statistics program R (v4.1.0) [32] with figures generated using the ggplot2 R package [33]. Communities were first rarefied down to 4000 ASV counts per sample (*rrarefy*; vegan package v2.5-7). Using this rarefied dataset, Shannon’s diversity [34] (*diversity*; vegan package v2.5-7) [35] and richness (sum of all ASVs in a sample) were calculated to obtain a measure of alpha diversity. Multivariate differences (e.g., beta-diversity) were measured by first transforming the rarefied dataset following a Hellinger transformation (*decostand*; vegan package v2.5-7), calculating a Bray-Curtis dissimilarity (*vegdist*; vegan package v2.5-7), and finally ordinated the data using a principal coordinate analysis (PCoA; *pcoa*; ape package v2.5-7) [36]. Phylum abundances were calculated by summing the rarified counts of all ASVs common to a given phylum.

### 2.5. Variables

#### 2.5.1. Outcome Variables

Microbial alpha-diversity (*Shannon Index*) and *Phylum Abundance*, which were calculated as described above, were the two outcome measures. The abundance of four phyla were used for analysis in this study: Firmicutes, Bacteroidetes, Proteobacteria, and Actinobacteria.

#### 2.5.2. Exposure Variables

The exposure variables were derived from frequency of culinary herb use data, which was surveyed during the INCLD Health study. The culinary herbs surveyed included cumin, garlic, onion, cinnamon, thyme, ginger, basil, rosemary, cilantro, parsley, sage, oregano, mint, dill, clove, cayenne, allspice, nutmeg, paprika, saffron, cardamom, tarragon, chives, bay leaf, coriander, red chili, black pepper, and fennel seed.

Frequency was reported in the survey as never, once per month, 2–3 times per month, once per week, twice per week, 3–4 times per week, 506 times per week, or daily. Frequency was recoded into low frequency (never to 2–3 times per month), medium frequency (once to twice per week), or high frequency (at least 3 times per week).

Dr. Dukes Phytochemical and Ethnobotanical Database [37] was used to identify chemical constituents of culinary herb, the parts per million (PPM) of each constituent, and which constituents had reported antibiotic properties. The Phenol-explorer 3.0 database [38,39] was then used to identify which chemical constituents were polyphenols. Following this identification, the culinary herbs were then grouped by certain phytochemical characteristics.

The *Alliums* group contained garlic, onions, chives, and the *Capsaicin* group contained chili pepper, paprika, and cayenne. Herbs containing at least 30,000 PPM of eugenol were grouped together (*Eugenol*) and included allspice, clove, and cinnamon. Two groups of herbs containing polyphenolic compounds were formed: A group with herbs containing over 50,000 PPM polyphenols *(>50,000 PPM Polyphenols*) included clove, cinnamon, fennel seed, thyme, oregano, onion, rosemary; a group with herbs containing over 30,000 PPM polyphenols *(>30,000 PPM Polyphenols*) contained those previously mentioned, as well as ginger, tarragon, cumin, basil, and allspice. Two groups of herbs containing compounds with reported antibiotic properties were formed: A group with herbs containing at least 90,000 PPM of compounds with reported antibiotic properties (>*90,000 PPM Antibiotic*) included clove, cinnamon, fennel seed, thyme, oregano, rosemary, black pepper, nutmeg, and cardamom; A group with herbs containing at least 30,000 PPM of compounds with reported antibiotic properties (>*30,000 PPM Antibiotic*) included those previously mentioned, as well as sage, bay leaf, mint, parsley, cumin, and allspice. The frequency of use of each of these herb groups (high, medium, and low frequency, as previously described) was then used as the exposure variables. Finally, the total number of herbs used with high frequency (*Total High Frequency Herbs*), defined as use at least 3–4 times per week, was used as an additional exposure variable. The final groups were not discrete, as there was some overlap between groups; however, each group was utilized in its own regression.

#### 2.5.3. Adjustment Variables

Adjustment measures included age, sex assigned at birth, race, and ethnicity, grouped as *Demographic Factors*; estimated daily intake of protein, fat, and fiber, grouped as *Dietary Factors*; the total number of supplements used, reported as *Total Supplements*; and the total number of medications used, reported as *Total Medication*.

Regarding demographic information, age was reported continuously; Sex assigned at birth was reported as male, female, or intersex; Ethnicity was reported as either Hispanic or Latino/Latina/LatinX, not Hispanic or Latino/Latina/LatinX, or unknown/not reported; Race was reported as Black or African American, Asian, Middle Eastern, Native Hawaiian or Pacific Islander, American Indian/Alaska Native, White/Caucasian, more than one race, or other/unknown. Any supplements and medications that were currently being used at the time of the baseline INCLD Health visit were reported and the total number of each were calculated. Estimated daily intake of protein, fat, and fiber were all reported in grams (g).

### 2.6. Statistical Models

To investigate the association between alpha-diversity and culinary herb use, linear regression models were used: Model 1 includes *Frequency of Use* of a specific culinary herb grouping variable, as described above; Model 2 adjusts for *Demographic Factors*; Model 3 adds adjustment for *Dietary Factors*; Model 4 adds adjustment for *Total Supplements;* Model 5 also adjusts for *Total Medication.* To investigate the association between phylum abundance and culinary herb use, the same Models 2–4 are used as described above.

Data was excluded from statistical analysis for the following purposes: (1) incomplete demographic data; (2) participants reported the combined use of a culinary herb with its matching supplement; for example, if a participant reported using turmeric as a culinary herb and reported using it as a supplement, that participant’s data was excluded from statistical analysis; (3) incomplete microbiome analysis data. All regression analyses were performed using SPSS Version 28.0.1.0 (IBM Corp, Armonk, NY, USA) [40].

## 3. Results

### 3.1. Descriptive Statistics

Average values for alpha diversity and phylum abundance are reported in Table 1. Baseline characteristics, regarding demographics, medications and supplements used, and intake of dietary factors, are reported in Table 2.

### 3.2. Frequency of Culinary Herb Use

**Figure 1:**
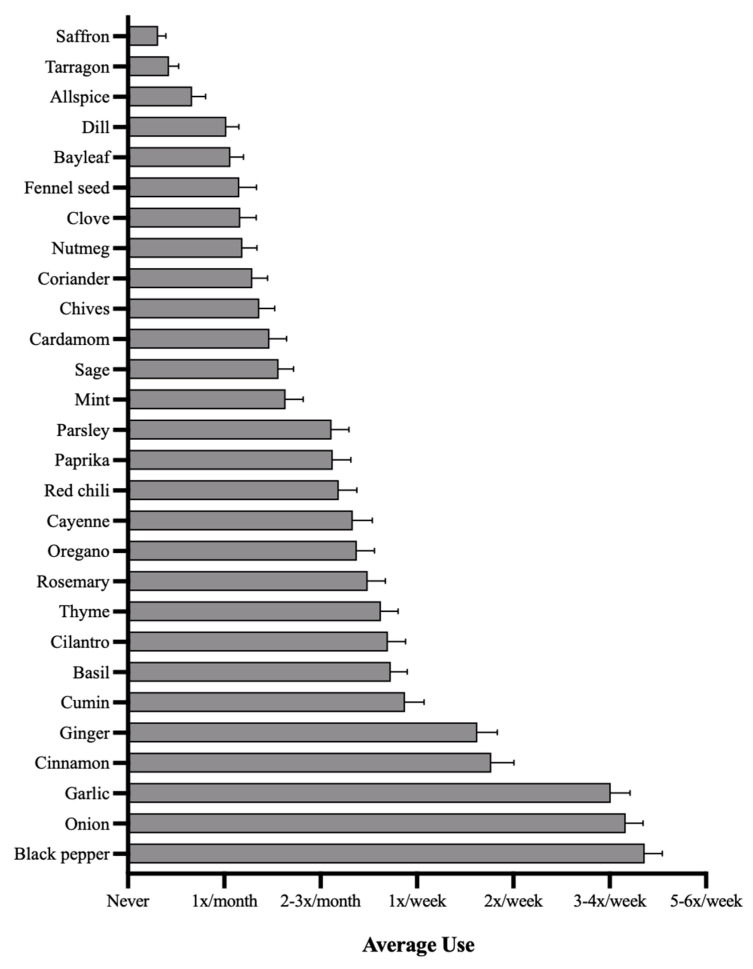
Average frequencies of culinary herb and spice use. Shown are the average frequencies of culinary herb and spice use with standard error bars, ranging from: Never, once per month, 2 to 3 times per month, once per week, twice per week, 3 to 4 times per week, and 5 to 6 times per week. No herbs averaged daily use. Herbs are displayed from least frequently used (top) to most frequently used (bottom).

**Figure 2:**
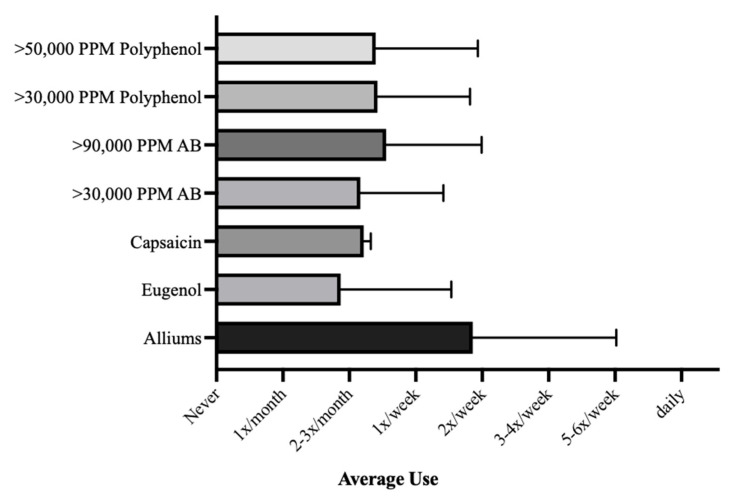
Average frequencies of culinary herb use by phytochemical grouping. Shown are the average frequencies of culinary herb and spice use with standard error bars, ranging from: Never, once per month, 2 to 3 times per month, once per week, twice per week, 3 to 4 times per week, and 5 to 6 times per week. No herb groups averaged daily use.

Black pepper was the most frequently used spice, being used on average about 4 times per week, followed by onion and garlic, which were used on average about 3 to 4 times per week (Figure 1). Cinnamon and ginger were the next most frequently used spices, being used between once to twice per week (Figure 1). All other spices were used on average less than once per week. No spice was used daily, on average (Figure 1). When considering the frequency of use of grouped herb categories, the *Alliums* group was the most frequently used, with an average use of about 2 times per week (Figure 2). Conversely, the *Eugenol* group was the least frequently used, with an average use of about 2–3 times per month (Figure 2).

### 3.3. Association between Frequency of Culinary Herb Use and Alpha Diversity

No statistically significant associations were observed between the frequency of culinary herb use and Shannon Index (Table 3). Additionally, there were no statistically significant associations observed between the total number of herbs used with high frequency and Shannon Index (Table 4).

### 3.4. Association between Frequency of Culinary Herb Use and Phylum Abundance

There were no significant associations observed between any of the exposure variables and Actinobacteria (Table 5) or Bacteroidota abundance (Table 6). However, a significant positive association was observed between Firmicute abundance and frequency of use of herbs in the >30,000 PPM antibiotic category, after adjusting for the total number of supplements and medications used (Table 7), and Firmicute abundance and the of use of herbs in the >90,000 PPM antibiotic category, independent of adjustments (Table 7). Additionally, significant positive associations were observed between Firmicute abundance and frequency of use of herbs in both the >30,000 PPM and >50,000 PPM polyphenol categories (Table 7), and between Firmicute abundance and the total number of herbs used with high frequency (Table 4), independent of adjustments.

Significant inverse associations were observed between Proteobacteria abundance and frequency of use for herbs the >30,000 PPM polyphenol category, after adjusting for dietary factors (Table 8); Non-significant inverse trends (*p*-values > 0.1) were observed in Models 1, 2, and 4. This same pattern was detected between Proteobacteria abundance and total number of herbs used with high frequency (Table 3). Significant inverse associations were also observed between Proteobacteria abundance and frequency of use for herbs the >50,000 PPM polyphenol category and >90,000 PPM antibiotic category, independent of adjustments (Table 8).

## 4. Discussion

This secondary data analysis of the INCLD Health cohort aimed to evaluate associations between the frequency of culinary herb use and microbiome diversity and phylum abundance. Our results indicate that the frequency of culinary herb use, particularly herbs with high phenolic content, as well as the total number of herbs used with high frequency, may be associated with changes in Firmicute and Proteobacteria abundance.

Polyphenols are metabolized by numerous gut microbes and thus act as prebiotics to certain taxa, such as commensal *Roseburia* spp., *Faecalibacterium* spp., *Lactobacillus* spp., which belong to the Firmicutes phylum [13]. While polyphenols can act as prebiotics for commensal bacteria, they many also interfere with virulence factors present in certain pathogenic bacteria, many of which are from the Proteobacteria phylum. For example, polyphenols from various sources decreased quorum sensing activity, motility, and biofilm formation of *Pseudomonas aeruginosa* [41,42]. Several polyphenols have also been shown to directly interfere with membrane-bound ATP synthase activity on *Escherichia coli* [43,44,45], thus disrupting energy production by these bacteria. It is possible these mechanisms may contribute to the associative patterns observed in this study, in which the frequency of high polyphenol-containing herb use was positively associated with Firmicute abundance and negatively associated with Proteobacteria abundance.

Clinical investigations into the impacts of polyphenol-based interventions further support the associative patterns observed in this study between high polyphenol-containing herbs and Firmicute and Proteobacteria abundance. When a polyphenol rich diet was administered to individuals with increased gut permeability [46], for example, serum metabolites of polyphenol catabolism were positively associated with bacteria in the order Clostridiales, as well as in the genera *Roseburia, Butyricicoccus and Faecalibacterium*, all of which are included in the Firmicutes phylum. Additionally, an inverse association was present between polyphenol-derived serum metabolites and the Proteobacteria of the genera *Desulfovibrio* and *Enterobacteriaceae* [46]. At the phylum level, a polyphenol-rich diet in individuals at high risk for cardiometabolic disorders resulted in increased Firmicutes abundance, as well an increase in members of the Clostridial cluster IV [47]. Similarly, a separate study observed that the administration of red wine polyphenols were associated with increases in Firmicutes phylum members, such as *Lactobacillus, Roseburia, Feacalibacterium,* and decrease in Proteobacteria phylum members, such as *Escherichia coli* and *Enterobacter* spp. [48], within fecal samples of study participants. Regarding the use of herbs specifically, independent of phenolic content, the administration of a multi-herb formula in adults with digestive disorders resulted in increased *Lactobacillus, Clostridium,* and *Faecalibacterium prausnitzii*; all members of the Firmicutes phylum [22].

A similar pattern of association was also observed between the frequency of herbs in the antibiotic categories, particularly those in the >90,000 PPM antibiotic category, and Firmicutes and Proteobacteria abundance. Studies looking specifically at the impact of select herbs on the gut microbiome reveal that some might perhaps reduce the abundance of opportunistic pathogens while increasing the abundance of commensal bacteria. Oregano, for example, contains numerous compounds reported to have antibiotic properties (thymol, carvacrol, p-cymene, y-terpinene, etc.) [37]. Oregano has been shown in vivo to reduce *Streptococcus* sp. and increase *Enterococcus* sp. when used in powdered form [49], and increase *Lactobacillus* sp., *Clostridium* sp., and the Firmicutes phylum when used in essential oil form [50]. Additionally, oregano use resulted in decreased opportunistically pathogenic members of the Proteobacteria phylum, such as the *Klebsiella* and *Proteus* genera [49]. Comparable results were observed with other herbs in the >90,000 PPM antibiotic category, or their constituents, demonstrating in previous studies an ability to increase commensal bacteria from the Firmicutes phylum and decrease opportunistically pathogenic bacteria from the Firmicutes and/or Proteobacteria phyla [19,51,52,53,54].

It is important to note, however, that there was overlap between the herbs in the antibiotic and polyphenol categories, as some herbs contained high quantities of both. Since these were not discrete categories, the observed positive association with Firmicute abundance may be an artifact of using herbs that also contain high quantities of polyphenols.

The pattern of positive association with Firmicute abundance, independent of adjustments, and inverse association with Proteobacteria abundance, after adjusting for dietary factors, was also observed for the total number of herbs used with high frequency. This category was originally included to investigate whether associations between frequency of culinary herb use and microbiome outcomes were due to the total number of different unique herbs used with high frequency, as opposed to specific biochemical profiles. Indeed, Macdonald et al. [55], previously described alpha diversity to be impacted by the consumption of 30 or more unique plants per week, regardless of plant type. However, due to the associative pattern shared with polyphenol-containing herb groups (i.e., associations with Firmicutes and Proteobacteria abundance, but not Shannon Index or the abundance of other phyla), we question whether the observed associations were impacted by participants consuming more herbs high in polyphenols when also using a great number of herbs with high frequency.

Finally, we did not observe associations between any of the exposure variables and the Shannon Index, a measure of alpha diversity. A recent randomized placebo-controlled trial investigating the effects of a daily measured dose of mixed culinary spices (cinnamon, oregano, ginger, black pepper, and cayenne pepper) on the gut microbiome also witnessed changes in the abundance of specific taxa, while not observing changes in alpha diversity [21]. At the phylum level, individuals taking the mixed spice intervention experienced an increase in the abundance of Firmicutes and a decrease in the abundance of Bacteroidetes. Despite detectable changes in the abundance of specific bacteria, however, alpha diversity and fecal SCFA concentrations, remained unchanged when compared to the placebo group [21]. Additionally, as previously mentioned, alpha diversity has been shown to be impacted by the consumption of 30 or more plant-based products per week, regardless of plant type [55]. As such, since the maximum total herbs used with high frequency by a participant was 17 herbs, it is possible participants did not reach the 30 plant per week threshold and/or consumed herbs in a large enough quantity to induce changes in diversity, even after adjusting for dietary factors. Moreover, it appears that our participants already entered the study with Shannon Index scores either above average or on the upper end of average, when compared to reported values from other studies. On average, our participants had an overall Shannon Index value of 4.13. While some previously reported values are between about 2 to 3.7 [56,57], others are more akin to our own observation, with values between 4 to 5 [58,59]. One study reporting an average Shannon Index of 4.63 in participants, which is similar to our own results, considered that value to be high [58].

### Strengths and Limitations

While phenolic compounds from various sources have previously been associated with changes in abundance of a variety of bacteria under both the Firmicutes and Proteobacteria phyla, this current study is the first to our knowledge which examines associations between microbiota and herbs with high phenolic content during typical culinary use. Additionally, the inclusion of medications, supplements, and dietary factors as adjustment variables, which are known to impact gut microbiota, allow for a detailed evaluation of how these factors might impact the associations between gut microbiota and culinary herb use.

This study, however, is not without limitations. The assessment of culinary herb use, which only considers frequency, leaves out other factors which may be important when considering how these herbs interact with the microbiome, such as herb preparation (e.g., fresh, dried) and quantity. Obtaining more detailed information on preparation and quantity could possibly allow for estimations of polyphenol ingestion.

Additionally, as diet provides numerous sources of polyphenols, the meaningful correlations observed could be a compounded effect of polyphenol-rich herbs adding to the polyphenol content already derived from other dietary sources (e.g., certain fruits and vegetables). An important future direction will be to estimate polyphenol content derived from non-herb dietary sources to determine how much, if any, it contributes to the associations observed.

Finally, due to the vastly disproportionate number of White/Caucasian participants, and the lack of robust representation of other racial and ethnic groups, a more granular statistical analysis of how such factors may impact the observed associations was not conducted. A larger sample size, coupled with community-based recruitment, as opposed to recruitment from a naturopathic college (i.e., the INCLD Health cohort), could provide a more diverse and generalizable sample for such an analysis. A larger sample size may also allow for a more detailed evaluation of bacterial abundance at the genus level as well as offer higher statistical power.

## 5. Conclusions

The current study provides evidence for associations between the frequency of culinary herb use and Firmicutes and Proteobacteria abundance at the phylum level, and that this relationship may be impacted by the phenolic content of culinary herbs. This novel insight supports further investigation into the role of culinary herbs in modulating the microbiome and may have future implications for dietary recommendations in the context of health and disease. As such, we intend to carry out a follow-up study, with a modified culinary herb use questionnaire, estimations of polyphenol intake, and a wider participant recruitment pool.

## Figures and Tables

**Table 1:** Characteristics of exposure and outcome variables (*n* = 96).

Variables	Value
Alpha Diversity	M (SD)
**Shannon Index**	29.34 (6.13)
**Phylum Abundance**	**M (SD)**
**Firmicutes**	2837.28 (398.21)
**Bacteroidota**	738.44 (309.27)
**Actinobacteria**	150.59 (172.71)
**Proteobacteria**	181.93 (243.70)
**Frequency of Herb Use**	**M (SD)**
**Allium**	3.86 (2.16)
**Eugenol**	1.87 (1.67)
**Capsaicin**	2.68 (1.42)
**>30,000 PPM Polyphenol**	2.43 (1.40)
**>50,000 PPM Polyphenol**	2.40 (1.54)
**>30,000 PPM Antibiotic**	2.07 (0.33)
**>90,000 PPM Antibiotic**	2.55 (1.44)

Shown are the average values for the frequency of herb use for each herb category used as an exposure variable, as well as the overall average value for Shannon Index and phylum abundance.

**Table 2:** Characteristics of study participants (*n* = 96).

Variable	Value
Age	M (SD)
	29.34 (6.13)
**Sex Assigned at Birth**	***n* (%)**
**Male**	14 (14.6%)
**Female**	81 (84.4%)
**Intersex**	1 (>1%)
**Race**	***n*** **(%)**
**White/Caucasian**	75 (78.1%)
**Asian**	5 (5.2%)
**African American**	2 (2%)
**Middle Eastern**	2 (2%)
**Native Hawaiian/Pacific** **Islander**	1 (1%)
**American Native/Alaska** **Native**	1 (1%)
**Mixed**	6 (6.3%)
**Other/Unknown**	4 (4.2%)
**Ethnicity**	***n* (%)**
**Hispanic/LatinX**	9 (9.4%)
**Non-Hispanic/LatinX**	83 (86.5%)
**Unknown**	4 (4.2%)
**Dietary Factors**	**M (SD)**
**Est. daily fat intake (g)**	80.6 (37.5)
**Est. daily protein intake (g)**	64.7 (30.7)
**Est. daily fiber intake (g)**	27.2 (10.8)
**Medications**	**M (SD)**
**Total Used**	0.6 (1.0)
**Supplements**	**M (SD)**
**Total Used**	5.7 (4.9)

Shown are the demographic factors (sex, age, race, ethnicity), dietary factors (estimated daily intake of fat, protein, and fiber in grams), and medication and supplement usage associated with study participants.

**Table 3:** Association between frequency of culinary herb use and Shannon Index.

Exposure:	Allium	Capsaicin	Eugenol	Antibiotic	Antibiotic	Polyphenol	Polyphenol
Freq. of Use				>30,000 PPM	>90,000 PPM	>30,000 PPM	>50,000 PPM
	β (CI)	β (CI)	β (CI)	β (CI)	β (CI)	β (CI)	β (CI)
	*p*-Value	*p*-Value	*p*-Value	*p*-Value	*p*-Value	*p*-Value	*p*-Value
**Model 1**	−0.067	0.009	0.015	0.079	0.136	0.056	−0.054
	(−0.068, 0.035)	(−0.063, 0.068)	(−0.054, 0.062)	(−0.037, 0.082)	(−0.021, 0.107)	(−0.047, 0.83)	(−0.076, 0.044)
	0.517	0.931	0.888	0.446	0.187	0.587	0.6
**Model 2**	−0.042	0.018	0.039	0.104	0.135	0.048	−0.042
	(−0.065, 0.043)	(−0.059, 0.071)	(−0.048, 0.070)	(−0.030, 0.090)	(−0.021, 0.106)	(−0.050, 0.082)	(−0.073, 0.049)
	0.696	0.858	0.709	0.32	0.189	0.646	0.69
**Model 3**	−0.055	−0.078	−0.048	0.063	0.109	0.044	−0.109
	(−0.068, 0.041)	(−0.094, 0.045)	(−0.076, 0.049)	(−0.047, 0.0.79)	(−0.031, 0.099)	(−0.057, 0.085)	(−0.098, 0.033)
	0.614	0.477	0.667	0.576	0.296	0.693	0.33
**Model 4**	−0.045	−0.094	−0.041	0.066	0.119	0.036	−0.109
	(−0.067, 0.044)	(−0.101, 0.041)	(−0.077, 0.054)	(−0.047, 0.063)	(−0.029, 0.104)	(−0.062, 0.085)	(−0.099, 0.035)
	0.688	0.403	0.725	0.564	0.266	0.754	0.342

Shown are the beta coefficients and *p*-values associated with each regression model. Model 1: Exposure variable (frequency of culinary herb use); Model 2: Model 1 + Demographic factors; Model 3: Model 2 + Dietary factors; Model 4: Model 3 + Total number of supplements and medications used.

**Table 4:** Association between total number of herbs used with high frequency and outcome variables.

Exposure:	Shannon Index	Firmicutes	Bacteroidota	Proteobacteria	Actinobacteria
Freq. of Use					
	β (CI)	β (CI)	β (CI)	β (CI)	β (CI)
	*p*-Value	*p*-Value	*p*-Value	*p*-Value	*p*-Value
**Model 1**	0.042	0.289	−0.154	−0.194	−0.037
	(−0.012, 0.018)	(11.2, 59.2)	(−33.8, 4.6)	(−29.5, 0.572)	(−12.8, 8.9)
	0.684	0.004 **	0.135	0.059	0.725
**Model 2**	0.046	0.286	−0.153	−0.198	−0.029
	(−0.012, 0.018)	(9.50, 59.7)	(−34.0, 5.8)	(−30.4, 0.940)	(−12.6, 9.0)
	0.66	0.007 **	0.152	0.064	0.783
**Model 3**	−0.022	0.301	−0.137	−0.235	−0.015
	(−0.018, 0.014)	(9.0, 63.9)	(−34.1, 9.8)	(−34.9, −0.590)	(−13.3, 10.5)
	0.842	0.009 **	0.243	0.044 *	0.897
**Model 4**	−0.021	0.294	−0.145	−0.216	−0.005
	(−0.018, 0.015)	(8.4, 62.2)	(−35.3, 9.5)	(−32.8, 0.462)	(−13.0, 11.2)
	0.855	0.009 **	0.223	0.055	0.968

Shown are the beta coefficients and *p*-values associated with each regression model. Model 1: Exposure variable (frequency of culinary herb use); Model 2: Model 1 + Demographic factors; Model 3: Model 2 + Dietary factors; Model 4: Model 3 + Total number of supplements and medications used. * *p*-value < 0.05; ** *p*-value < 0.01.

**Table 5:** Association between frequency of culinary herb use and Actinobacteria abundance.

Exposure:	Allium	Capsaicin	Eugenol	Antibiotic	Antibiotic	Polyphenol	Polyphenol
Freq. of Use				>30,00 PPM	>30,000 PPM	>90,000 PPM	>50,000 PPM
	β (CI)	β (CI)	β (CI)	β (CI)	β (CI)	β (CI)	β (CI)
	*p*-Value	*p*-Value	*p*-Value	*p*-Value	*p*-Value	*p*-Value	*p*-Value
**Model 1**	−0.044	−0.017	−0.033	−0.077	−0.102	−0.119	0.032
	(−46.61, 30.06)	(−52.64, 44.46)	(−48.98, 36.55)	(−60.72, 27.69)	(−71.35, 24.04)	(−77.05, 20.34)	(−37.79, 51.77)
	0.669	0.867	0.773	0.46	0.327	0.251	0.757
**Model 2**	0.01	−0.015	0.039	−0.03	−0.107	−0.09	0.059
	(−37.12, 40.80)	(−50.68, 43.68)	(−35.09, 51.13)	(−50.23, 37.31)	(−71.48, 21.53)	(−69.94, 26.03)	(−31.25, 57.07)
	0.926	0.883	0.713	0.771	0.289	0.379	0.563
**Model 3**	−0.001	0.01	0.071	−0.031	−0.112	−0.12	0.072
	(−40.83, 40.40)	(−49.57, 54.39)	(−32.31, 61.68)	(−55.23, 41.75)	(−74.56, 22.32)	(−83.90, 21.70)	(−33.30, 64.43)
	0.992	0.927	0.536	0.783	0.287	0.287	0.528
**Model 4**	−0.002	0.024	0.095	−0.033	−0.105	−0.106	0.083
	(−41.70, 40.99)	(−47.70, 58.99)	(−29.78, 69.31)	(−55.46, 42.46)	(−74.38, 25.54)	(−82.87, 26.36)	(−31.77, 67.81)
	0.986	0.834	0.43	0.792	0.334	0.36	0.474

Shown are the beta coefficients and *p*-values associated with each regression model. Model 1: Exposure variable (frequency of culinary herb use); Model 2: Model 1 + Demographic factors; Model 3: Model 2 + Dietary factors; Model 4: Model 3 + Total number of supplements and medications used.

**Table 6:** Association between frequency of culinary herb use and Bacteroidota abundance.

Exposure:	Allium	Capsaicin	Eugenol	Antibiotic	Antibiotic	Polyphenol	Polyphenol
Freq. of Use				>30,000 PPM	>90,000 PPM	>30,000 PPM	>50,000 PPM
	β (CI)	β (CI)	β (CI)	β (CI)	β (CI)	β (CI)	β (CI)
	*p*-Value	*p*-Value	*p*-Value	*p*-Value	*p*-Value	*p*-Value	*p*-Value
**Model 1**	−0.067	−0.164	−0.046	−0.135	−0.087	−0.13	−0.067
	(−81.07, 56.26)	(−155.98, 16.58)	(−93.79, 59.26)	(−126.90, 30.64)	(−121.66, 49.39)	(−142.59, 31.56)	(−106.24, 53.87)
	0.517	0.113	0.655	0.191	0.404	0.209	0.518
**Model 2**	−0.042	−0.171	−0.054	−0.124	−0.074	−0.125	−0.061
	(−92.79, 51.68)	(−158.38, 14.22)	(−100.00, 60.02)	(−132.96, 28.18)	(−117.66, 55.68)	(−141.26, 36.43)	(−105.67, 58.35)
	0.696	0.101	0.621	0.253	0.497	0.238	0.568
**Model 3**	−0.055	−0.157	−0.015	−0.106	−0.053	−0.118	−0.025
	(−89.54, 61.65)	(−161.90, 29.66)	(−93.28, 82.15)	(−136.83, 42.75)	(−112.84, 68.56)	(−145.72, 51.50)	(−101.10, 81.31)
	0.614	0.174	0.9	0.364	0.629	0.31	0.83
**Model 4**	−0.045	−0.16	−0.032	−0.128	−0.064	−0.127	−0.032
	(−93.48, 60.67)	(−165.91, 31.08)	(−104.71, 80.83)	(−138.61, 43.00)	(−120.34, 66.80)	(−152.21, 51.70)	(−105.68, 80.64)
	0.688	0.177	0.799	0.298	0.571	0.292	0.79

Shown are the beta coefficients and *p*-values associated with each regression model. Model 1: Exposure variable (frequency of culinary herb use); Model 2: Model 1 + Demographic factors; Model 3: Model 2 + Dietary factors; Model 4: Model 3 + Total number of supplements and medications used.

**Table 7:** Association between frequency of culinary herb use and Firmicute abundance.

Exposure:	Allium.	Capsaicin	Eugenol	Antibiotic	Antibiotic	Polyphenol	Polyphenol
Freq. of Use				>30,000 PPM	>90,000 PPM	>30,000 PPM	>50,000 PPM
	β (CI)	β (CI)	β (CI)	β (CI)	β (CI)	β (CI)	β (CI)
	*p*-Value	*p*-Value	*p*-Value	*p*-Value	*p*-Value	*p*-Value	*p*-Value
**Model 1**	0.113	0.195	0.127	0.198	0.347	0.255	0.279
	(−39.3, 136.6)	(−3.5, 216.1)	(−37.2, 158.5)	(−1.7, 198.7)	(78.2, 275.0)	(26.3, 214.7)	(44.8, 261.9)
	0.275	0.058	0.221	0.054	0.001 **	0.013 *	0.006 **
**Model 2**	0.106	0.198	0.114	0.192	0.342	0.247	0.272
	(−48.2, 139.2)	(−4.3, 219.2)	(−48.8, 158.4)	(−8.5, 199.3)	(71.4, 276.6)	(19.1, 214.4)	(37.4, 261.8)
	0.337	0.059	0.296	0.071	0.001 **	0.020 *	0.010 *
**Model 3**	0.101	0.221	0.084	0.193	0.33	0.284	0.327
	(−53.9, 140.6)	(−18.3, 227.7)	(−72.6, 153.3)	(−4.3, 224.2)	(61.8, 274.4)	(27.6, 241.2)	(57.7, 301.8)
	0.378	0.059	0.479	0.094	0.002 **	0.014 *	0.004 **
**Model 4**	0.131	0.142	0.089	0.225	0.302	0.266	0.286
	(−38.7, 151.0)	(−44.9, 199.6)	(−72.2, 157.1)	(0.86, 222.2)	(46.0, 262.1)	(18.4, 233.2)	(34.6, 279.5)
	0.242	0.212	0.463	0.048 *	0.006 **	0.022 *	0.013 *

Shown are the beta coefficients and *p*-values associated with each regression model. Model 1: Exposure variable (frequency of culinary herb use); Model 2: Model 1 + Demographic factors; Model 3: Model 2 + Dietary factors; Model 4: Model 3 + Total number of supplements and medications used. * *p*-value < 0.05; ** *p*-value < 0.01.

**Table 8:** Association between frequency of culinary herb use and Proteobacteria abundance.

Exposure:	Allium	Capsaicin	Eugenol	Antibiotic	Antibiotic	Polyphenol	Polyphenol
Freq. of Use				>30,000 PPM	>90,000 PPM	>30,000 PPM	>50,000 PPM
	β (CI)	β (CI)	β (CI)	β (CI)	β (CI)	β (CI)	β (CI)
	*p*-Value	*p*-Value	*p*-Value	*p*-Value	*p*-Value	*p*-Value	*p*-Value
**Model 1**	−0.057	−0.091	−0.102	−0.141	−0.347	−0.193	−0.258
	(−69.05, 39.07)	(−98.67, 37.79)	(−89.96, 30.15)	(−104.81, 19.05)	(−143.73, −19.82)	(−132.80, 2.99)	(−140.21, −18.03)
	0.583	0.378	0.325	0.072	0.010 **	0.061	0.012 *
**Model 2**	−0.037	−0.087	−0.099	−0.137	−0.342	−0.203	−0.262
	(−67.29, 47.57)	(−98.39, 40.25)	(−92.27, 34.40)	(−105.61, 22.36)	(−147.92, −19.74)	(−137.90, 1.36)	(−143.44, −17.42)
	0.734	0.407	0.366	0.199	0.011 *	0.055	0.013 *
**Model 3**	−0.038	−0.11	−0.113	−0.189	−0.227	−0.257	−0.326
	(−70.01, 50.09)	(−113.10, 39.90)	(−120.49, 36.14)	(−128.23, 13.17)	(−152.76, −19.71)	(−164.08, −11.1)	(−169.30, −30.88)
	0.742	0.344	0.344	0.109	0.012 *	0.025 *	0.005 **
**Model 4**	−0.063	−0.048	−0.098	−0.191	−0.225	−0.201	−0.311
	(−74.68, 41.29)	(−90.86, 59.93)	(−98.41, 40.78)	(−125.60, 9.73)	(−137.05, −3.03)	(−143.45, 8.16)	(−162.44, −28.34)
	0.568	0.673	0.413	0.092	0.041 *	0.078	0.006 **

Shown are the beta coefficients and *p*-values associated with each regression model. Model 1: Exposure variable (frequency of culinary herb use); Model 2: Model 1 + Demographic factors; Model 3: Model 2 + Dietary factors; Model 4: Model 3 + Total number of supplements and medications used. * *p*-value < 0.05; ** *p*-value < 0.01.

## Data Availability

The data presented in this study are available within this article.
